# Association of *5-HT2C* (rs3813929) and *UCP3* (rs1800849) gene polymorphisms with type 2 diabetes in obese women candidates for bariatric surgery

**DOI:** 10.1590/2359-3997000000260

**Published:** 2017-03-20

**Authors:** Noa Pereira Prada Schnor, Rozangela Verlengia, Patrícia Fátima Sousa Novais, Alex Harley Crisp, Celso Vieira de Souza Leite, Irineu Rasera, Maria Rita Marques de Oliveira

**Affiliations:** 1 Universidade Estadual Paulista Júlio de Mesquita Filho Araraquara SP Brasil Universidade Estadual Paulista Júlio de Mesquita Filho (Unesp), Programa de Pós-Graduação em Alimentos e Nutrição, Araraquara, SP, Brasil; 2 Universidade Metodista de Piracicaba Piracicaba SP Brasil Universidade Metodista de Piracicaba (Unimep), Programa de Pós-Graduação em Ciências do Movimento Humano, Piracicaba, SP, Brasil; 3 Unesp Faculdade de Medicina Botucatu SP Brasil Unesp, Faculdade de Medicina, Botucatu, SP, Brasil; 4 Clínica Bariátrica Piracicaba SP Brasil Clínica Bariátrica, Piracicaba, SP, Brasil

**Keywords:** Obesity, type 2 diabetes, hypertension, gene polymorphism

## Abstract

**Objective:**

Obesity can cause systemic arterial hypertension (SAH) and type 2 diabetes mellitus (DM2) factor that is also influenced by genetic variability. The present study aims to investigate the association between gene polymorphisms related with obesity on the prevalence of SAH and DM2 in the preoperative period and 1 year after Roux-en-Y gastric bypass surgery.

**Subjects and methods:**

In total, 351 obese women in a Brazilian cohort completed the study. The clinical diagnosis of SAH and DM2 was monitored from medical records. Twelve gene polymorphisms (rs26802; rs572169; rs7799039; rs1137101; rs3813929; rs659366; rs660339; rs1800849; rs7498665; rs35874116; rs9701796; and rs9939609) were determined using real-time polymerase chain reaction and TaqMan assay.

**Results:**

In the preoperative period, prevalence of SAH and DM2 was 57% and 22%, respectively. One year postoperatively, 86.8% subjects had remission of DM2 and 99.5% had control of SAH. Subjects with T allele from the serotonin receptor gene (5-HT2C, rs3813929) had five times greater chance of DM2, and the CC genotype from uncoupling protein 3 gene (UCP3, rs1800849) had three times greater chance in the preoperative period.

**Conclusion:**

These findings indicate that polymorphisms rs3813929 and rs1800849 from 5-HT2C and UCP3 genes were related to DM2 prevalence among the Brazilian obese women candidates for bariatric surgery.

## INTRODUCTION

Obesity prevalence is increasing at a worrying rate; with significant influence on morbidity and mortality rate worldwide (1). Excessive food intake and a sedentary lifestyle contribute to the accumulation of body fat and increase the risk of chronic diseases such as systemic arterial hypertension (SAH) and type 2 diabetes mellitus (DM^2^) (2). Moreover, genetic factors are recognized as an important influence in increasing susceptibility to obesity and its associated comorbidities (3).

A genetic factor of considerable interest is the variation of a gene (DNA sequence bases) between subjects, described as gene polymorphism (4). In this context, some studies have related polymorphism of specifics genes that control appetite, energy metabolism, susceptibility to obesity (5), and chronic diseases such as DM2 and SAH (6).

It is recognized that obesity and its associated comorbidities have multifactorial causes. Therefore, it is important to analyze several genes and the interaction between them to better understand the obesity genesis and the contribution of genetic variability. Review studies indicate a large number of gene candidates that may be related to obesity and chronic diseases (7-9).

Regarding the treatment of obesity, bariatric surgery is the most long-term effective method of inducing body weight loss and beneficial effects on metabolic disorders (10). However, there is a significant variability in body weight loss and control of comorbidities between subjects undergoing bariatric surgery (11). This suggests that some of these variable responses to bariatric surgery can be explained by genetic factors.

In this sense, investigating the relationship between uncoupling protein 3 (*UCP3*) gene polymorphism (rs1800849) and results after biliopancreatic diversion surgery (BDS), Luis and cols. (12) observed no significant association with the control of SAH and DM2 1 year postoperatively. However, another study from Luis and cols. (13) indicated that fat mass and obesity-associated (*FTO*, rs9939609) gene polymorphism were associated with greater body weight loss 3 months after BDS, but no difference was evident after 9 and 12 months postoperatively. In addition, Luis and cols. (13) observed an association between *FTO* gene polymorphism (rs9939609) with a decrease in the body mass index (BMI), and improvements of glycated hemoglobin (HbA1c) levels 6 months after mini gastric bypass laparoscopic surgery. Therefore, other studies are warranted to investigate a larger number of gene polymorphisms to expand the evidence regarding the effect of genetic variability as a determinant of SAH and DM2 on obesity and bariatric surgery response.

The main benefit of surgical treatment of obesity is the control of comorbidities. Therefore, the current study selected 12 gene polymorphisms related to obesity in order to assess an association with SAH and DM2 prevalence, and the possible influence on bariatric surgery results. There is some evidence that suggests effects on the prevalence of DM2 with polymorphisms of ghrelin (*GHRL*, rs26802), uncoupling protein 2 (*UCP2*, rs659366), uncoupling protein 3 (*UCP3*, rs1800849), *FTO* (rs9939609), leptin (*LEP*, rs7799039), leptin receptor (*LEPR*, rs1137101), and serotonin receptor (*5-HT2C*, rs3813929) genes.

Thus, the current study aimed to investigate the association of 12 gene polymorphisms on the prevalence of SAH and DM2 in the preoperative period and 1 year after Roux-en-Y gastric bypass (RYGB) surgery in obese women.

## SUBJECTS AND METHODS

This is a prospective study with adult women undergoing RYGB surgery, performed by the same medical staff from June 2010 to May 2013. The subjects were evaluated in the preoperative period and 1 year postoperatively. Self-reported information regarding age, skin color, education, previous pregnancies, family history of obesity, and obesity at early age was recorded. Body weight, BMI, and the presence of SAH and DM2 were provided by medical records.

The eligible criteria for participation were: (a) being female; (b) aged between 20 and 50 years; (c) and registered on the waiting line for bariatric surgery. The exclusion criteria were as follows: (a) alcoholism; (b) genetic syndromes associated with obesity; (c) Cushing’s syndrome; (d) hypothyroidism; (e) renal or liver failure; (f) neoplasia; (g) infection with human immunodeficiency virus (HIV); (h) use of corticosteroids; (i) and postmenopausal women using estrogen replacement.

In total, 441 women in a Brazilian cohort were eligible for study inclusion. However, subjects who did not attend 1 year postoperatively were not included in data analysis. This study included 351 subjects who completed the study. The number of subjects was determined using the data of the genotype with lower prevalence and a minimum 300 subjects was shown to be necessary. All subjects signed a free-and-informed consent form after being informed about the procedures involved in the research. This study was approved by the local Research Ethics Committee (protocol number: 3303/2009).

Body weight was measured using a digital balance (Fizola, SP, Brazil) with a capacity of 200 kg, precise to within 100 g. Height measurements were obtained using a stadiometer (Seca, SP, Brazil) precise to within 0.1 cm. The procedures followed the standard recommendations (14). Excess weight (EW, in kg); weight loss (WL, in kg), and percentage of excess weight loss (%EWL), were calculated using the followings formulas (15):

EW = body weight preoperatively – ideal body weight;

WL = body weight preoperatively – current body weight;

%EWL = percentage of body weight lost in relation to excess weight.

The SAH remission was considered when the subject stopped with anti-hypertensive medications and presented: systolic blood pressure < 140 mmHg and diastolic blood pressure < 90 mmHg (16). The DM2 remission was considered when the subject stopped with anti-diabetic medications and presented: fasting glucose concentration < 126 mg/dL; postprandial glucose < 200 mg/dL after 75g oral glucose load, and hemogloblin A1c level < 6.5% (17).

Genomic DNA was isolated from whole blood samples (EDTA-treated), using Illusta blood genomicPrep Kit (GE Healthcare^®^, New York, USA). The analysis of gene polymorphisms *GHRL* (rs26802); *GHRS* (rs572169); *LEP* (rs7799039); *LEPR* (rs1137101); *5HT2C* (rs3813929); *UCP2* (rs659366); *UCP2* (rs660339); *UCP3* (rs1800849); *SH2B1* (rs7498665); *TAS1R2* (rs35874116); *TAS1R2* (rs9701796); and *FTO* (rs9939609), was determined by real-time polymerase chain reaction (RT-PCR) and TaqMan assay (Applied Biosystems^®^, Branchburg, New Jersey, USA). The RT-PCR was processed in ABI 7500 fast equipment (Applied Biosystems^®^, Branchburg, NJ, USA) according to the manufacturer’s instructions. A random selection of 10% of the samples was again genotyped to evaluate the reproducibility of genotyping.

The agreement of genotype frequencies with Hardy–Weinberg equilibrium expectations was tested by chi-square test. Multiple logistic regressions were performed to evaluate the effects of gene polymorphism (with adjustments for age, skin color, preoperative BMI, previous pregnancy, and age at onset of obesity) taking SAH and DM2 as dependent variables. When the genotype of lower frequency was < 10%, the genotype was combined with a heterozygous genotype. *p* < 0.05 was considered to be statistically significant.

## RESULTS

The genotype distributions of gene polymorphisms (rs26802; rs572169; rs7799039; rs1137101; rs3813929; rs659366; rs660339; rs1800849; rs7498665; rs35874116; rs9701796; rs9939609) was within the expectations of the Hardy-Weinberg equilibrium (*p* > 0.05). Table 1 shows characterization of the study subject.

**Table 1:** Characteristics of subject study

Variables	Median (min–max)
Age (years)	34 (20–50)
Age at onset of obesity (years)	18 (0–40)
Preoperative BMI (kg/m^2^)	46 (33–73)
BMI postoperatively (kg/m^2^)	30 (20–55)
%EWL 1 year postoperatively	69 (37–119)
DM2 preoperative – n (%)	
Yes	76 (22)
No	275 (88)
DM2 1 year postoperative – n (%)	
Yes	10 (2.8)
No	341 (97.2)
SAH preoperative – n (%)	
Yes	201 (57)
No	150 (43)
SAH 1 year postoperative – n (%)	
Yes	01 (0.3)
No	350 (99.7)
Family history of obesity – n (%)	
Yes	279 (79.5)
No	72 (20.5)
Skin Color – n (%)	
Black	52 (14.8)
White	226 (64.4)
Parda	73 (20.8)
Education – n (%)	
Middle and High School (incomplete)	123 (35)
High School (complete) and superior	228 (65)
Previous pregnancies – n (%)	
Yes	263 (74.9)
No	88 (25.1)

*n* = 351. BMI: body mass index; %EWL: percentage of excess weight loss.

One year postoperatively, of 76 subjects with DM2, only 10 maintained the diagnosis of DM2, representing a control of 86.8%. Regarding SAH, 99.5% of subjects had control of this disease. The median of %EWL was 69.2%; of which 4.3% of the subjects had less than 50% of %EWL and 26.7% had DM2. One subject did not have control of SAH postoperatively and presented 58.7% of %EWL.

Because of a high proportion of control of these comorbidities postoperatively, logistic regression analysis was possible only with preoperative data. Among 12 gene polymorphisms analyzed, *5HT2C* (rs3813929) and *UCP3* (rs1800849) had influence on DM2 prevalence. In the *5HT2C* gene, subject carriers of the T allele had a five times greater chance of DM2 compared to CC genotype. In *UCP3* gene, CC genotype had three times greater chance of DM2 compared with T allele carriers (Table 2). However, there was no prevalence of *5-HT2C* (*rs3813929*) and *UCP3* (*rs1800849*) gene polymorphisms among diabetic subjects who obtained or did not obtain the control of DM2 postoperatively (Table 3).

**Table 2:** Values of odds ratio (OR) and 95% confidence interval (95% CI) for diabetes mellitus type 2 (DM2) and systemic arterial hypertension (SAH) before and after bariatric surgery, according to each gene polymorphism

Gene Polymorphism	Genotype	n (%)	DM2 Preoperative		SAH Preoperative	

			OR (95% CI)	p-value	OR (95% CI)	p-value
*GHRL rs26802*	CC	35 (10)	1.00	0.921	1.00	0.563
	CA + AA	316 (90)	1.045 (0.436–2.507)		1.244 (0.593–2.609)	
*GHSR rs572169*	GG	211 (60)	1.00	0.547	1.00	0.443
	GA + AA	140 (40)	0.722 (0.250–2.085)		1.441 (0.566–3.667)	
*LEP rs7799039*	AA	53 (15)	1.00	0.318	1.00	0.274
	AG + GG	298 (85)	0.652 (0.282–1.508)		0.712 (0.388–1.307)	
*LEPR rs1137101*	GG	73 (21)	1.00	0.974	1.00	0.945
	AG + AA	278 (79)	0.989 (0.514–1.902)		1.019 (0.596–1.742)	
*5–HT2C rs3813929*	TT/CT	87 (25)	1.00	**0.001**	1.00	0.556
	CC	264 (75)	0.251 (0.109–0.578)		1.164 (0.702–1.928)	
*UCP2 rs659366*	TT	49 (14)	1.00	0.505	1.00	0.884
	CT + CC	302 (86)	1.330 (0.574–3.082)		1.056 (0.511–2.181)	
*UCP2 rs660339*	AA	49 (14)	1.00	0.238	1.00	0.675
	AG + GG	302 (86)	0.568 (0.222–1.452)		1.167 (0.567–2.405)	
*UCP3 rs1800849*	CC	54 (15)	1.00	**0.019**	1.00	0.128
	CT + TT	297 (85)	0.305 (0.113–0.821)		0.624 (0.341–1.144)	
*SH2B1 rs7498665*	GG	223 (64)	1.00	0.594	1.00	0.946
	AG + AA	294 (84)	0.815 (0.385–1.726)		0.980 (0.544–1.765)	
*TAS1R2 rs35874116*	CC + CT	184 (52)	1.00	0.790	1.00	0.933
	TT	167 (48)	1.706 (0.626–1.850)		1.019 (0.658–1.579)	
*TAS1R2 rs9701796*	GG + CG	117 (33)	1.00	0.353	1.00	0.341
	CC	234 (67)	0.761 (0.428–1.354)		1.252 (0.788–1.989)	
*FTO rs9939609*	AA	71 (20)	1.00	0.911	1.00	0.058
	AT + TT	280 (80)	0.963 (0.492–1.882)		0.595 (0.348–1.017)	

n = 351. Variables with adjustments for age, skin color, preoperative BMI, previous pregnancy and age at onset of obesity.

**Table 3:** Prevalence of gene polymorphism for *5–HT2C (rs3813929)* and *UCP3 (rs1800849)* between diabetic subjects who obtained or did not obtain the control of DM2 after bariatric surgery

Response	CT + CC	CT + CT	CT + TT	CC + CC	CC + CT	CC + TT
Yes	1/65	8/58	4/62	7/59	22/44	24/42
No	0/10	1/9	1/9	1/9	3/7	4/6
p-value*	0.868	1.000	0.985	1.000	1.000	0.999

* Fisher’s exact test.

## DISCUSSION

This study investigated the association of 12 gene polymorphisms related to obesity on the prevalence of SAH and DM2 during the preoperative period and 1 year after RYGB surgery in obese women. Our main finding indicated that *5-HT2C* (rs3813929) and *UCP3* (rs1800849) gene polymorphisms were associated with the prevalence of DM2 in obese women candidates for bariatric surgery.

This knowledge might be important in identifying, according to genetic variability, subjects who are at higher risk of DM2, because it is not obesity per se that is the lethal factor, but it’s associated chronic diseases. Thus, the analysis of *5-HT2C* (rs3813929) and *UCP3* (rs1800849) gene polymorphism may theoretically define individualized and preventive measures for DM2 in obese women on a waiting list for bariatric surgery.

Unlike the results of other studies (18-23), the current study did not find an association of *GHRL* (rs26802), *UCP2* (rs659366), *FTO* (rs9939609), *LEP* (rs7799039), and *LEPR* (rs1137101) gene polymorphisms with DM2. These controversies, far from discouraging the study of the effects of gene polymorphisms, instigate a deepening of this knowledge, which takes into account the characteristics of the populations and the interactions between the different gene polymorphisms.

Regarding *5-HT2C* gene polymorphism results, serotonin plays an important role in the nervous system, such as sleep regulation, body temperature, appetite and mood, among others (24). Thus, low levels of serotonin or problems in the signaling with the receptor have been linked to an increased desire to eat sweets and carbohydrates (25), which may explain in part the association of *5-HT2C* gene polymorphism (rs3813929) with the prevalence of DM2 observed in our study.

Similarly, other studies reported that subjects carrying the T allele from *5-HT2C* gene polymorphism (rs3813929) were associated with greater chances of DM2. Kring and cols. (26), investigating Caucasian young men, observed an association of T allele from *5-HT2C* gene polymorphism with glucose and acute insulin response. On the other hand, Iordanidou and cols. (27) observed that T allele frequency was lower in diabetic subjects compared to nondiabetic subjects.

In the present study, the *UCP3* gene was also associated with prevalence of DM2, specifically the polymorphism rs1800849. The *UCP3* protein is involved in promoting fatty acid oxidation in skeletal muscle and it indirectly influences glucose metabolism (28). In addition, *UCP3* regulates the production of reactive oxygen species in the mitochondria (28), a factor that has established an association of this protein with DM2, because oxidative stress pathways play a key role in the development of this chronic disease (29).

In this study, women carrying the CC genotype on *UCP3* gene polymorphism (rs1800849) showed three times more chance of DM2 compared to allele T carriers. Corroborating our data, Meirhaeghe and cols. (30) showed that *UCP3* gene polymorphism (rs1800849) was related to DM2 on a French cohort, and T allele carriers presented lower risk.

Regarding data 1 year postoperatively, the 12 gene polymorphisms investigated in our study showed no effect on the control/remission of SAH and DM2. Among the diabetic women in this study, 13% did not obtain the control of this chronic disease; thus, it is plausible to hypothesize that there are a possible genetic factors involved in the lack of this response postoperatively. On the other hand, considering that 1 year postoperatively is a relatively short time period, it is important to do a long-term investigation (> 2 years) to determine these responses.

Although the molecular and genetic comprehension of DM2 has advanced rapidly, much of the knowledge remains unknown. Adding to this, the number of bariatric surgeries has risen as an effective treatment option for obesity and its associated comorbidities. Thus, more studies are necessary to investigate the effects of surgical and non-surgical intervention for weight loss and remission of DM2, while larger numbers of subjects, a wider range of gene polymorphism, and analyzing a later postoperative time, could better elucidate these questions.

Genetic association studies are relevant to determine the risk of chronic diseases on a specific population and to improve the medical and nutritional treatment. These results enable an individualization of the treatment according to genetic variability. The current study investigates 12 obesity-related gene polymorphisms in obese women candidates for bariatric surgery.

In summary, subjects with T allele from *5-HT2C* gene polymorphism (rs3813929) had five times greater chance of DM2, and the CC genotype from *UCP3* gene polymorphism (rs1800849) had three times greater chance of DM2, in the preoperative period. Our results indicate that *5-HT2C* and *UCP3* gene polymorphism was related to prevalence of DM2 among Brazilian obese women candidates for bariatric surgery.
